# Artificial Intelligence in Cardiac Electrophysiology: A Comprehensive Review

**DOI:** 10.3390/jpm15110532

**Published:** 2025-11-03

**Authors:** Pietro Cipollone, Nicola Pierucci, Andrea Matteucci, Marta Palombi, Domenico Laviola, Raffaele Bruti, Sara Vinciullo, Marco Bernardi, Luigi Spadafora, Angelica Cersosimo, Sara Trivigno, Tommaso Recchioni, Agostino Piro, Cristina Chimenti, Claudio Pandozi, Carmine Dario Vizza, Carlo Lavalle, Marco Valerio Mariani

**Affiliations:** 1 Department of Cardiovascular, Respiratory, Nephrological, Aenesthesiological and Geriatric Sciences “Sapienza”, University of Rome, 00161 Rome, Italy; pietro.cipollone@uniroma1.it (P.C.); domenico.laviola@uniroma1.it (D.L.); raffaelemaria.bruti@gmail.com (R.B.); sara.vinciullo@uniroma1.it (S.V.); sara.trivigno@uniroma1.it (S.T.); tommaso.recchioni@uniroma1.it (T.R.); agostino.piro@uniroma1.it (A.P.); cristina.chimenti@uniroma1.it (C.C.); dario.vizza@uniroma1.it (C.D.V.); carlo.lavalle@uniroma1.it (C.L.); marcovalerio.mariani@uniroma1.it (M.V.M.); 2 Clinical and Rehabilitation Cardiology Division, San Filippo Neri Hospital, 00135 Rome, Italy; andrea.matteucci2@gmail.com (A.M.); cpandozi@libero.it (C.P.); 3 Department of Medical-Surgical Sciences and Biotechnologies, Sapienza University of Rome, 00185 Latina, Italy; marco.bernardi23@gmail.com (M.B.); luigi.spadafora@uniroma1.it (L.S.); 4 Division of Cardiology and Department of Medical and Surgical Specialties, Radiological Sciences and Public Health, University of Brescia, 25121 Brescia, Italy; angelica.cersosimo@gmail.com

**Keywords:** atrial fibrillation, personalized medicine, electroanatomical mapping

## Abstract

**Background:** Artificial Intelligence (AI) is a transformative innovation designed to enable machines to perform tasks typically requiring human intelligence. Among various medical fields, cardiology—and particularly electrophysiology—has seen rapid integration of AI technologies. The ability of AI to analyze large and complex datasets is reshaping diagnostic and therapeutic approaches. **Objectives:** This review aims to provide a comprehensive overview of AI models and their applications in cardiac electrophysiology. The focus is on understanding how AI contributes to clinical practice through ECG interpretation, arrhythmia detection, atrial mapping, and catheter ablation, while also exploring its limitations and future potential. **Methods**: The review discusses various AI approaches, including Machine Learning (ML) and Deep Learning (DL), and highlights relevant literature illustrating their implementation in electrophysiological settings. Key clinical applications are examined thematically, with a narrative synthesis of current capabilities, technologies, and outcomes. **Results**: AI-based tools have demonstrated effectiveness in identifying supraventricular arrhythmias like atrial fibrillation (AF) and atrial flutter (AFL), as well as complex conditions such as ventricular tachycardias (VTs) and long QT syndrome (LQTS). In procedural contexts, AI enhances electro-anatomical mapping, reduces operative time, and supports tailored post-ablation management. **Discussion**: While AI offers clear advantages in diagnostic accuracy and procedural efficiency, challenges remain regarding data security, ethical transparency, and clinical adoption. Addressing these limitations will be crucial for integrating AI into routine electrophysiology and maximizing its potential in future cardiology practice.

## 1. Overview of Artificial Intelligence

### 1.1. Defining Artificial Intelligence

AI can be defined as the field of computer science dedicated to developing systems and algorithms capable of performing tasks that typically require human intelligence [1]. Within AI, which encompasses a wide range of concepts, various models and systems have been designed to analyze data using different rules, allowing their application to diverse tasks.

Initially, the quality of the data provided to AI software was manually curated by human operators, who also imposed predefined “rules” governing how the software associated and processed the data. In this way, machines were able to generate autonomous reasoning based on these fundamental rules.

Artificial Intelligence (AI) is one of the most important technological innovations with a lot of applications across numerous fields, including finance, education, and, most notably, medicine. When AI first emerged in the mid-20th century, it was primarily an academic endeavor, largely restricted to symbolic reasoning and rule-based systems. Over time, with increased computational power, availability of large-scale datasets, and advancements in neural network architectures, AI systems have become indispensable tools in healthcare [2,3,4]. From automated image interpretation to predictive analytics that anticipate clinical outcomes, AI techniques now permeate many facets of modern medical practice [5].

Cardiology and electrophysiology (EP) are one of the earliest medical disciplines in which AI has been used. While traditional EP methods have been highly refined through decades of clinical practice, AI presents opportunities to enhance diagnostic accuracy, reduce procedural times, and improve long term patient outcomes [1].

The purpose of this review is to provide a comprehensive overview of AI methods and their use in EP and analyze the key areas of cardiology where AI is making a difference, with particular emphasis on ECG interpretation, atrial mapping, and catheter ablation (CA). Additionally, the review will discuss challenges, ethical considerations, and likely future directions of AI in EP.

### 1.2. Machine Learning

The term “machine learning” (ML) was introduced over 70 years ago to define the capability of artificial systems to acquire the ability to execute complex tasks and enhance their performance through accumulated experience [6]. ML is a widely used AI model in medicine and has had a great impact on cardiology. ML relies on algorithms that analyze large datasets (such as patient profiles, sensor data, or imaging results) and associate them with a set of desired outputs, including diagnoses, risk scores, and clinical decisions [5].

The degree of human intervention in ML can vary significantly, and based on the extent and quality of this intervention, different ML models can be identified.

#### 1.2.1. Supervised Learning

In supervised learning (SL), the dataset contains labeled examples. For instance, an algorithm can be trained to detect atrial fibrillation (AF) by analyzing thousands of ECG segments already labeled as either “AF” or “normal.” This can be achieved using a binary approach (distinguishing between normal and abnormal ECGs) or a multiclass approach (categorizing the specific type of rhythm, such as normal, atrial fibrillation, or atrioventricular block, among others) [5]. By learning from these labeled cases, the system establishes decision boundaries and can classify new ECG data accordingly. One of the most The bold formatting was removed where not necessaryused SL algorithms is k-nearest neighbors (KNN). This is a classification algorithm based on similarity. Decision-making is based on identifying and analyzing feature patterns in the existing database that closely resemble those of the input currently under evaluation. SL is particularly powerful in clinical applications where accuracy (diagnosing an arrhythmia) is critical.

#### 1.2.2. Unsupervised Learning

Unsupervised learning works with datasets that lack explicit labels. Instead, the algorithm looks for hidden patterns and relationships, such as clustering patients with similar profiles or identifying underlying structures in high-dimensional data [5]. In cardiology, unsupervised learning is useful for discovering subgroups of heart failure patients who may respond differently to specific treatments, paving the way for more personalized medicine.

#### 1.2.3. Reinforcement Learning

Reinforcement learning takes a different approach; it interacts with the environment by taking actions and receiving feedback in the form of rewards or penalties. Over time, the algorithm learns to maximize positive outcomes. While often associated with robotics and game-playing AI, reinforcement learning holds great promise for healthcare applications. For example, it could be used to dynamically adjust the dosage of antiarrhythmic drugs, optimizing treatment effectiveness while minimizing side effects [7].

### 1.3. Deep Learning

Deep Learning (DL), a more advanced and complex extension of ML, is a data-driven modeling approach designed to recognize patterns in data and generate predictions. This technology has significantly influenced various aspects of modern life, enabling voice commands on smartphones and facilitating personalized advertising [8]. DL is composed of multilayered neural networks called deep neural network (DNN). A DNN is a particular type of artificial neural network that is inspired, in a simplified way, by the functioning of the human brain. This system is composed of many layers of artificial neurons that progressively transform the input information to reach a final decision. They are called “deep” precisely because the information passes through numerous levels before emerging as an output.

**Figure 1:**
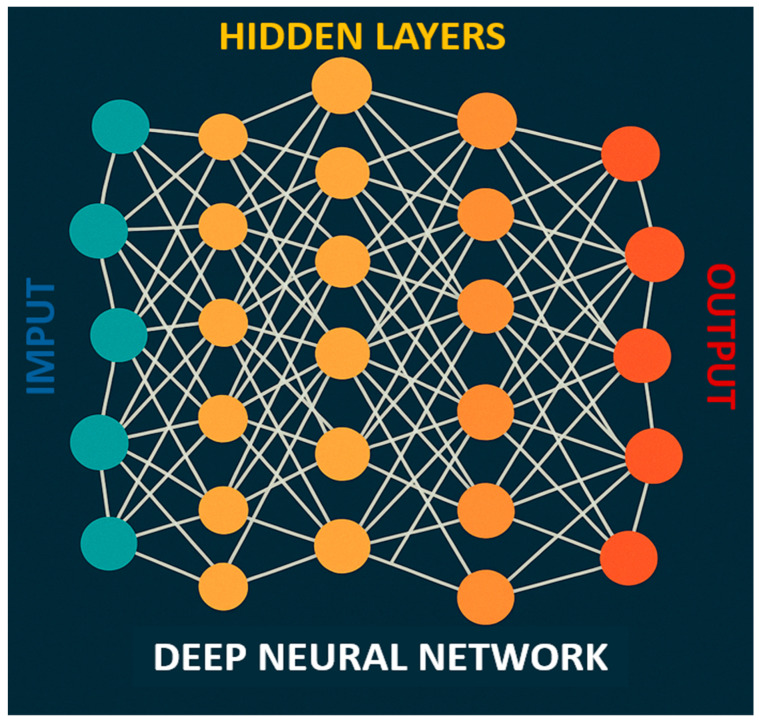
A DNN processes raw data by passing it through an input layer, followed by multiple hidden layers. Each neuron connection has a “weight” adjusted during training to improve performance. Neurons perform basic mathematical operations, but their internal workings are not directly interpretable (black boxes). The network’s power comes from combining numerous calculations on complex data, ultimately producing the final output in the output layer. DNN: deep neural network.

The functioning of a DNN starts with raw data, such as an image or an ECG trace, that is fed into the first layer of the network, called the input layer. From there, the information passes through a series of intermediate layers, known as hidden layers, and each connection between neurons is assigned a “weight” which is adjusted during the training process to optimize the model’s performance. Each node (or neuron) in these layers performs simple mathematical operations, and the operations carried out by each neuron are not directly accessible or controllable by the user, which is why such systems are often referred to as “black boxes”. The power of the network arises from the combination of millions of these calculations on complex data. At the end of this process, the output layer produces the final result, which may be a prediction, a classification, or another type of response (Figure 1).

Although deep learning requires vast amounts of data and significant computational power, it has demonstrated exceptional capabilities in tasks such as speech recognition, image generation, and image classification.

The functioning of a DL model can be designed to be more suited for certain types of tasks than others. As a result, different DL architectures have been developed, each optimized for specific applications (Table 1):

- Convolutional Neural Networks (CNNs): Particularly well-suited for image processing but also increasingly applied to time series data (like ECG waveforms), CNNs have been employed for automated segmentation of cardiac structures in imaging, detection of ischemic regions, and classification of arrhythmias from ECGs signals [9].
- Recurrent Neural Networks (RNNs) and Long Short-Term Memory (LSTM) Networks: These networks handle sequential data, making them ideally suited for analyzing ECG signals, which are inherently temporal. LSTM networks address the vanishing gradient problem that plagued early RNNs, allowing them to capture longer dependencies across a time series.

### 1.4. Computational Model and AI

Beyond AI, recent scientific literature has increasingly focused on the development of computational models (CM) capable of faithfully capturing the complexity of human physiology [10,11]. CM appears particularly well-suited to address this need. A CM is a mathematical or algorithmic abstraction of a real-world biological system, designed to simulate, predict, and provide deeper insight into its behavior. These models leverage equations, algorithms, and large datasets to represent complex physiological phenomena that are often inaccessible to direct observation. In medicine, and particularly in cardiology, CMs have been employed to replicate cardiac anatomy and function, hemodynamic flow, electrical conduction, and even population-level responses to therapeutic interventions. The integration of computational modeling, which inherently reflects the intricate dynamics of the cardiovascular system, with the analytical power of AI, holds the potential to profoundly shape the future landscape of scientific research in medicine, with cardiology poised to be one of its principal beneficiaries.

**Table 1:** Different types of AI architecture compared.

Architecture	Full Name	Best For	Core Components	Strengths	Limitations
ANN	Artificial Neural Network	General classification and regression tasks	Fully connected layers	Simple and general-purpose	Poor at capturing spatial/temporal patterns
CNN	Convolutional Neural Network	Image processing, computer vision	Convolutional and pooling layers	Captures spatial features, fewer parameters	Struggles with sequential data
RNN	Recurrent Neural Network	Sequential data (e.g., time series, text)	Recurrent connections	Good for time-dependent patterns	Short memory, vanishing gradient problem
LSTM	Long Short-Term Memory	Long-term sequence modeling	Memory cells with gates	Handles long dependencies, avoids vanishing gradients	More complex, slower training
GAN	Generative Adversarial Network	Image synthesis, data augmentation	Generator and discriminator networks	Generates realistic data, creative tasks	Training instability, mode collapse
Autoencoder	Autoencoder	Dimensionality reduction, denoising	Encoder and decoder networks	Efficient data compression and reconstruction	May underfit, hard to interpret results

## 2. Artificial Intelligence in Electrophysiology

Among the various branches of cardiology, EP is undoubtedly the field in which AI is most widely utilized. Advanced AI models have been developed for preclinical or in vitro studies, for the automated analysis of ECGs, enabling the diagnosis not only of arrhythmias but also of potential ischemic events. Additionally, AI has been integrated into atrial and ventricular mapping systems, enhancing precision and efficiency in electrophysiological assessments (Table 2).

### 2.1. Artificial Intelligence in In Vitro and Cellular Studies

Electro-anatomical properties and their alterations in pathological substrates form the cornerstone of research in cardiac electrophysiology and are essential for understanding the genesis of arrhythmias and for informing therapeutic strategies. Currently, the study of the electro-anatomical characteristics of the cardiac conduction system is limited by the invasiveness of existing diagnostic techniques. In this context, AI may represent a significant innovation, potentially revitalizing research efforts in the field. In 2021, Jeong and Lim [12] proposed an artificial neural network model capable of predicting changes in cardiac ion channel conductance by analyzing simulated action potential (AP) morphologies. Their findings suggest that ML-based analysis of AP waveforms may offer a noninvasive alternative to conventional, more complex approaches for identifying specific ion channel dysfunctions. Similarly, Cantwell et al. [35] and Sánchez et al. [13] explored the application of AI, particularly ML, for the analysis of the anatomical substrate in patients with atrial fibrillation (AF), including data acquired in vitro. Their work demonstrated the ability of AI models to interpret complex signals and provide more reliable classification of arrhythmogenic sites, highlighting the strong correlation between microstructural and macrostructural substrates. Collectively, these studies suggest that AI-based technologies could represent a breakthrough in the personalization of electrophysiological therapy.

### 2.2. Artificial Intelligence and ECG Interpretation

ECG interpretation has historically relied on visual inspection by clinicians. Since digitalization of ECG, AI methods have been employed in computerized interpretation of ECGs [8].

In recent decades, thanks to the increased use of wearable devices capable of monitoring heart rate over varying durations (implantable loop recorder, smart watch, life vest) [36,37], the clinical data that doctors must manage has increased significantly. In this scenario, AI can deeply help during the analysis process and guide clinicians toward the most appropriate diagnostic and therapeutic pathway.

As explained by Galloway et al. [38], AI has the potential to identify electrolyte abnormalities such as hyperkalemia. Early and effective recognition of electrolyte disorders could help clinicians to manage patients undergoing high diuretic therapy.

AI is a powerful tool, and as stated by Attia et al. [6], it can be used to expand the potential of the electrocardiogram, a diagnostic instrument that has been a key clinical tool for many years. AI can transform ECG analysis into a powerful screening and predictive tool for both cardiac and non-cardiac diseases, often in asymptomatic patients.

Through DNN, AI has demonstrated remarkable capabilities in detection of left ventricular dysfunction, specifically reduced ejection fraction, with a high-accuracy AUC > 0.90; diagnosis of hypertrophic cardiomyopathy, even in the absence of overt ECG abnormalities; screening for valvular diseases; and prediction of non-cardiac conditions, such as liver cirrhosis, by recognizing subtle ECG alterations [4].

Randomized clinical trials involving AI have also been conducted [38], highlighting both the analytical capabilities of AI and its integration into controlled study designs. Lin et al. [39] evaluated the impact of an AI-enabled ECGs analysis system in identifying hospitalized patients at high risk of mortality. Their study, which included over 15,000 patients across multiple hospitals, demonstrated that implementation of the AI system led to a significant reduction in 90-day all-cause mortality (3.6% vs. 4.3%; hazard ratio [HR] = 0.83).

AI-driven ECG analysis has the potential to enhance early diagnosis, improve clinical decision-making, and expand the role of this noninvasive modern medicine [6].

With experience, cardiologists and electrophysiologists can discern subtle anomalies; however, human-based interpretation remains subject to variability and requires substantial time and expertise. The application of AI, especially CNNs, has revolutionized ECG analysis, achieving near or even surpassing expert level performance.

### 2.3. Artificial Intelligence and Arrhythmias Detection

One of the first applications of AI was ECG analysis. In recent decades, devices have been developed that are able to analyze ECGs and recognize heart rhythm abnormalities, in some cases being able to identify the specific type of rhythm disorder.

The study conducted by Hannun et al. [14] describes the development and validation of a DNN designed for automated detection and classification of cardiac arrhythmias using single-lead ambulatory electrocardiogram data. The dataset used for training consisted of single-lead ambulatory ECGs with an average monitoring duration of approximately 10.6 days per patient.

The DNN was trained to classify 12 distinct rhythm classes and demonstrated excellent diagnostic performance, achieving an average area under the receiver operating characteristic curve (AUC) of 0.97. Notably, the DNN achieved an average F1 score of 0.837, exceeding the average cardiologist F1 score of 0.780. When specificity was fixed at the average cardiologist specificity, the model’s sensitivity surpassed the average cardiologist sensitivity across all rhythm classes.

Misclassifications by the DNN closely mirrored those of expert cardiologists, highlighting realistic clinical complexities encountered in ECG interpretation. The model performed exceptionally well for clinically significant arrhythmias such as atrial fibrillation, atrioventricular block, and ventricular tachycardia. These results underline the potential clinical utility of deep learning in automated arrhythmia diagnosis, suggesting that such models could significantly reduce diagnostic errors, improve diagnostic accuracy, and enhance clinical workflow efficiency.

### 2.4. Artificial Intelligence and Atrial Fibrillation Detection

AF is certainly one of the most frequent arrhythmias that cardiologists meet during their daily activity. AI could aid in the detention of AF and help cardiologists but also non-cardiologists in the detention of AF. AI models capable of recognizing AF have been developed by several research groups in recent years.

Some studies have focused on developing AI models that can identify AF through 12-lead ECG analysis and be able to detect early development of AF starting from SR ECG.

The group of Attia et al. [15] trained a CNN to identify the electrocardiographic signature of AF on standard 10 s, 12-lead ECGs recorded at the Mayo Clinic from 1994 to 2017. The dataset included 649.931 ECGs, allocated into training (454.789 ECGs), internal validation (64.340 ECGs), and testing (130.802 ECGs) subsets. The AI model detected AF with AUC of 0.87, a sensitivity of 79.0%, a specificity of 79.5%, and an accuracy of 79.4%. When incorporating multiple ECGs per patient within a 31-day window, performance improved, achieving an AUC of 0.90, sensitivity of 82.3%, specificity of 83.4%, and an accuracy of 83.3.

The group of Yuan et al. [16] has assessed a deep learning model’s effectiveness, using CNN, to predict the presence of AF within 31 days from outpatient 12-lead ECGs in sinus rhythm. ECG data from 1987 to 2022, collected from six US Veterans Affairs (VA) hospitals and one external academic center (Cedars-Sinai), were analyzed. The study involved 907,858 sinus rhythm ECGs. The DL model trained on data from VA San Francisco and Palo Alto sites demonstrated robust predictive performance on withheld test datasets, achieving AUC values ranging from 0.88 to 0.89 and an accuracy of approximately 81–82%. On an external test dataset from Cedars-Sinai Medical Center, the model’s predictive ability improved, showing an AUC of 0.93 and accuracy of 87%.

Instead, the group of Beak et al. [17] developed an AI-based algorithm to detect subtle differences in paroxysmal AF (PAF) during SR. The AI-model was trained with 241.212 lead-ECGs (70% for training, 10% for validation, and 20% for testing). The model showed excellent ability in PAF identification in internal and external validation, respectively, with an AUC of 0.79 and 0.75 and F1 score of 75% and 74%.

In addition, Kurshid et al. [18] trained a CNN to infer 5-year AF risk using 12-lead ECGs. The authors trained the CNN model with 45.770 patients and validated it on 83.162 patients form two different datasets (Brigham and Women’s Hospital and UK Biobank). Then, they assessed the efficiency of the AI-model with clinical risk score (CHARGE-AF). The CNN-based model has similar predictive usefulness of a clinical risk score and may enable efficient quantification of AF risk in daily clinical practice.

In addition, Hygrell et al. [19] assessed an AI-based model designed to predict paroxysmal AF from single-lead sinus rhythm electrocardiograms. The AI model was developed using 478,963 ECG recordings from 14,831 patients aged 65 years or older, collected during three screening studies (SAFER, STROKESTOP I, and STROKESTOP II). ECGs from 80% of patients from SAFER and STROKESTOP II were used for training, while ECGs from the remaining 20% of these two studies and the entire STROKESTOP I cohort formed the test set. The AI-based CNN achieved an AUC of 0.80 in the SAFER study, which had a broader age distribution (65–90+ years). In contrast, performance was lower in the age-homogenous cohorts of STROKESTOP I and STROKESTOP II (age range 75–76 years), with AUC of 0.62 in both studies. These studies have shown that the use of artificial intelligence can be very useful in the detention of AF, with an accuracy comparable to that of experienced cardiologists. This approach offers the potential to enable earlier identification and management of individuals at risk, even in the absence of overt arrhythmic manifestations.

Over the past decades, the advent of novel technologies has facilitated the increasing dissemination of wearable and implantable single-lead diagnostic devices, fundamentally transforming the management of patients experiencing arrhythmic events. These devices generate substantial volumes of data; the application of AI-based algorithms to such datasets holds the potential to significantly enhance both the speed and accuracy of arrhythmia diagnosis. Several studies have focused on the development of AI models capable of detecting atrial fibrillation (AF) from single-lead electrocardiograms (ECGs). Furthermore, groups of Mittal et al. [20] and Sarkar et al. [21] have provided compelling evidence that the integration of AI-based algorithms into implantable loop recorders significantly improves their diagnostic performance in detecting episodes of atrial fibrillation (AF), notably by reducing the incidence of false-positive detections.

In addition, groups of Noseworthy et al. [22] and Dupulthys et al. [23] focused their studies on wearable devices (WD) that are increasingly adopted due to their ease of use and reliability. Although they are highly valuable in clinical practice, they generate such a large volume of data that their routine management can become burdensome for clinicians. The integration of AI technologies could enhance the efficiency of these devices, making them more effective and easier to use. These studies demonstrates that a targeted screening strategy powered by AI and based on existing clinical data was shown to increase the detection yield of atrial fibrillation, offering the potential to enhance the overall efficacy of AF screening initiatives.

### 2.5. Artificial Intelligence and Ventricular Tachycardia Detection

Ventricular tachycardia (VTs) and premature ventricular contractions (PVCs) are a challenge for cardiologists. The analysis of ventricular tachycardia ECGs can often lead to errors, even for the most experienced cardiologist. The localization of the focus of VT or PVC is the prerogative of the most experienced electrophysiologist who, from the analysis of the surface ECG, can identify the exact location of the focus harboring the arrhythmia. The ability to precisely locate the focus is of considerable importance for planning a possible transcatheter ablation procedure. The AI can be helpful in analyzing surface ECGs and recognizing the focus responsible for ventricular arrhythmia.

Yu et al. [24] introduced a novel approach for automatic detection of PVCs, using deep metric learning and k-nearest neighbors (KNN) classifier. Accurate identification of PVCs is clinically relevant as this arrhythmia, although often asymptomatic, can be associated with serious heart conditions. Traditionally, analyzing long-term ECG data acquired from wearable devices such as Holter monitors is time-consuming for cardiologists. The proposed method automatically extracts essential features from heartbeats through a deep metric learning neural network. This network learns feature representations that minimize intraclass variance and maximize interclass differences, thereby facilitating classification. The KNN classifier uses these learned features to categorize heartbeats based on their distances. This methodology simplifies signal analysis by eliminating complex preprocessing steps and avoids manual feature engineering, thus reducing potential biases.

Validation was performed using the standard MIT-BIH database, widely recognized for benchmarking similar studies, achieving outstanding results: an accuracy of 99.7%, sensitivity of 97.45%, and specificity of 99.87%.

Another study by Missel et al. [25] introduced an innovative hybrid ML approach to localize the origin of ventricular tachycardia (VT) using 12-lead ECGs. Initially, a population-based DL model trained on general patient data provides a preliminary localization of VT origin. Then, a patient-specific model incrementally refines this localization by actively guiding clinicians in real time to optimal pacing sites during procedures. This patient-specific model progressively improves its predictions with each added pacing data, significantly enhancing accuracy and efficiency. The presented hybrid method outperforms traditional approaches based only on clinical or morphological data, indicating its potential for significantly improving the efficiency and precision of ventricular tachycardia ablation site localization.

These outcomes demonstrate the reliability and effectiveness of the proposed methods, positioning it competitively against existing traditional morphological and deep learning methods. These new models will also allow cardiologists who are not specialized in electrophysiology to recognize more rapidly and thus manage complex arrhythmias such as VT, which have always been the exclusive competence of the electrophysiologist.

### 2.6. Artificial Intelligence and Long QT Detection

Congenital long QT syndrome (LQTS) is characterized by the prolongation of the QT interval in 12-lead ECG and may manifest as syncope, convulsions or sudden cardiac death. Although a QTc 500 ms increases the risk of events, about 40% of patients have normal QTc at rest (LQTS hidden) [40]. In addition to QTc, other ECG parameters and stress tests improve diagnosis and risk stratification. In the management of LQTS AI can be a valuable help.

Bos et al. [26] evaluated the effectiveness of CNN to identify genetically confirmed patients with LQTS, even in cases with an apparently normal QT interval (<450 ms). The study analyzed 12-lead electrocardiograms from 2059 patients, 967 patients with confirmed LQTS and 1092 patients assessed but discarded as normal, for a total of 9085 ECGs. The CNN model was trained using 60% of ECG, validated in 10%, and tested on the remaining 30%. The model was very effective in calculating QTc with an AUC of 0.8. In addition, the AI approach was able to differentiate effectively between the three main genetic subtypes of LQTS (LQT1, LQT2 and LQT3), reaching a maximum AUC of 0.944.

Instead, Jiang et al. [27] explored the application of DL using CNN for screening and differentiating congenital long QT syndrome (LQTS) and its two major genetic subtypes (LQT1 and LQT2) using 12-lead ECG data. A total of 4521 ECGs from 990 patients were analyzed. CNN demonstrated high diagnostic performance with an AUC of 0.93 for LQTS detection and an AUC of 0.91 for genotype differentiation. The CNN method significantly outperformed expert measured QTc intervals, achieving a higher sensitivity (0.90 vs. 0.36) in identifying LQTS, particularly in patients with normal or borderline QT intervals, known as concealed LQTS (sensitivity 0.78).

In another study, Giudicessi et al. [28] trained and validated an AI-enabled 12-lead ECG algorithm to determinate QTc. The algorithm was trained using 1.6 million of ECGs from 538,200 patients (250,767 patients for training, 107,920 patients for testing, and 179,513 were used for validation) to calculate the QTc using “gold standard” cardiologist-overread QTc values. Then, the DNN algorithm was tested on 686 patients with genetic heart disease using ECG derived both from 12-lead ECG and a single-lead ECG obtained from a prototype device (AliveCor KardiaMobile 6L (AliveCor, Inc., Mountain View, CA, USA)). The QTc calculated from the DNN algorithm and those calculated from experts were very similar, with poor difference (−1.76 ± 23.14 ms). When applied to single-lead ECG, the sensitivity and specificity to detect QTc > 500 ms were 80% and 94,4%, respectively.

These findings suggest that DL-driven models, in particular CNN, can significantly enhance the detection and genetic classification of LQTS from resting ECGs, supporting its potential clinical application for improving diagnosis accuracy and clinical management in challenging diagnostic scenarios.

### 2.7. AI and Sudden Cardiac Death

Implantable cardioverter defibrillators (ICDs) are widely used for primary prevention of sudden cardiac death (SCD); many patients who receive ICDs never require therapy during the battery lifespan, and many SCD events occur in individuals considered low risk by conventional criteria. AI could help cardiologists with risk stratification and consequently optimize ICD assignment.

Popescu et al. [29] created a DL-based algorithm that blends neural networks and magnetic resonance (MR) imaging to predict the risk of SCD in patients with ischemic heart disease. The algorithm was evaluated on multi-center internal validation data and tested on another independent set of patients. The DL-based algorithm achieved concordance index of 0.83 and 0.74 and 10-year integrated Brier score of 0.12 and 0.14. These results show that the DL system can estimate, in a very accurate way, risk of SCD from clinical information and MR imaging.

Instead, Barker et al., in their review [41], analyzed 11 studies. A total of 6 of these 11 studies compared normal risk stratification models with AI-based models; they observed that 5 of these showed that AI-based models were superior to traditional scores; the AUC of AI-based models ranged from 0.71 to 0.96 across studies.

However, the review identified significant methodological limitations, including incomplete adherence to reporting standards and high risk of bias in five studies. Despite these issues, the authors concluded that ML holds promise for enhancing SCD prediction compared to traditional models, primarily due to its capacity to integrate and analyze complex multidimensional datasets. They also emphasize the need for developing and adhering to specialized reporting standards to enhance transparency and reproducibility in future ML studies.

### 2.8. Artificial Intelligence and Catheter Ablation

Atrial mapping is critical in the evaluation and treatment of arrhythmias like AF, atrial flutter, and other complex tachycardias. Physicians rely on electroanatomic mappings to locate abnormal conduction pathways or scar tissue that serve as the substrate for arrhythmogenesis. High-density mapping systems provide detailed electrical measurements, but the volume of data can be enormous and difficult to interpret manually.

AI algorithms can rapidly process high-density signals, highlighting regions of interest and offering data-driven insights into arrhythmia mechanisms [2,42]. By combining mapping data with imaging modalities such as magnetic resonance imaging (MRI) or computed tomography (CT), AI can create a unified representation of both structural and electrical abnormalities.

The study by Seitz et al. [30] evaluated VX1, an AI-based software developed by Volta Medical, aimed at standardizing electrogram-based ablation procedures for persistent AF. Persistent AF is challenging due to variability in interpreting complex electrograms. VX1 employs multiparametric machine-learning algorithms, trained on a large database of expert-annotated electrograms, to automatically generate real-time dispersion maps. These maps visually highlight abnormal electrogram regions, guiding targeted ablation and reducing operator-dependent variability. This prospective multicenter study involved 85 patients treated by 17 operators across eight centers. Ablation guided by VX1 resulted in acute AF termination in 88% of cases, with direct conversion to sinus rhythm achieved in 65%. Follow-up after approximately 13.5 months showed freedom from documented AF in 86% after a single procedure, rising to 89% after an average of 1.3 procedures per patient. Importantly, freedom from any atrial arrhythmia significantly improved from 54% after one procedure to 73% after repeat procedures (*p* < 0.001).

Outcomes were consistent between the primary and satellite centers, demonstrating that AI effectively standardizes complex ablation procedures across different settings. Moreover, VX1 accurately identified all critical ablation sites where AF termination or significant cycle length prolongation occurred. When compared to a historical group receiving visually guided dispersion mapping, VX1 achieved comparable clinical efficacy.

To help electrophysiologists during ablative procedures, Biosense Webster (Biosense Webster, Irvine, CA, USA) recently introduced a DL-based software (CARTOSOUND FAM Module), enabling anatomical reconstruction of the left atrium (LA) from intracardiac echocardiography (ICE) images [31].

Di Biase et al. enrolled 28 patients undergoing AF ablation and compared the automatic reconstruction of LA anatomical structures, obtained through ICE integrated with the DL algorithm, with images acquired by cardiac computed tomography (CT), considered the gold standard. The pulmonary vein ostia diameters and the distance between venous carinae were comparable between the two techniques, showing moderate-to-high correlation for most structures, except for the right inferior pulmonary vein (RIPV).

Qualitative evaluation performed by three independent electrophysiologists confirmed good agreement between images generated by the algorithm and those obtained by CT. The average anatomical reconstruction time using the algorithm was approximately 65 s. All patients successfully underwent ablation without immediate complications.

AI-based models can be used, also, to predict the success of an ablative procedure or the risk of AF recurrence after an CA procedure. Razeghi et al. [32] presented an AI-based model for predicting outcomes of AF ablation. The approach utilizes a machine learning fusion framework that integrates cardiac computed tomography (CT)-derived morphological features, patient-specific clinical data, and deep learning from raw CT images.

This combined approach significantly improves the predictive capability over traditional methods that rely solely on clinical or morphological data.

Instead, two interesting studies [33,34] have used AI-based models to effectively identify predisposing factors for AF recurrence. This approach allows for each patient a personalized path, promoting patient adherence to medical care and improving therapeutic outcomes.

In conclusion, the AI-based VX1 software, CARTOSOUND FAM, and the AI-based models effectively standardize and optimize selection of patients, as well as electroanatomical mapping and ablation, improving procedural consistency and clinical outcomes in AF treatment, supporting its potential integration into routine electrophysiology practice.

## 3. Challenges and Limitations

The implementation of AI in clinical practice would undoubtedly offer substantial advantages, particularly regarding the analysis of extensive datasets. However, several critical issues would concurrently require careful consideration.

### 3.1. Data Quality and Generalizability of Analyses

The studies cited in this review often employ clinical data collected retrospectively, pertaining to populations that, despite sometimes being extensive, remain limited by ethnic and geographical variables. Consequently, the developed models may lack full generalizability. AI algorithms necessitate large, high-quality datasets for reliable training. The presence of inconsistent data, missing values, and variability in acquisition protocols may negatively impact model performance and introduce bias.

### 3.2. Transparency and Data Privacy

AI, particularly DL, utilizes neural networks, characterized by continuous and intricate algorithmic interactions that complicate control of each logical step leading to the model’s conclusions. Each step of the process appears opaque and inaccessible, resembling a black box in which the internal mechanisms are not readily interpretable. This complexity constitutes a significant limitation when entrusting AI models with highly sensitive clinical data. Furthermore, ensuring patient privacy is challenging, especially in contexts involving automated data sharing. Enhancing the security, privacy, and reliability of AI systems would significantly improve their effectiveness and applicability in daily clinical practice. Additionally, specific informed consent protocols must be developed and clearly presented to patients. Here, medical and healthcare professionals play a pivotal role in comprehensively and understandably explaining the processes and benefits associated with AI use. An increasing number of experts in the field are questioning how to make neural networks more transparent and black boxes more accessible [43]. Alternatively, some authors argue that attempting to explain every single interaction is not an effective way to address the problem. Instead, they suggest that it is preferable to start with an explainable AI model when dealing with high-impact decisions [44]. Unlike conventional AI approaches, particularly deep learning, XAI aims not only to deliver accurate decision-making but also to provide transparent and understandable explanations for the model’s outputs, thereby enhancing trust and interpretability in critical applications [45]. Numerous proposals have been put forward, and it is likely that, soon, innovative solutions will emerge to help dispel the uncertainty that still surrounds neural networks and artificial intelligence today.

### 3.3. Accessibility

The use of AI relies on complex mathematical models that require dedicated software and hardware. The widespread implementation of AI in routine clinical practice must be accompanied by adequate dissemination and accessibility of advanced technologies capable of supporting the data flow and computational capacity demanded by AI.

### 3.4. Human Oversight

AI leverages sophisticated algorithms and exceptionally efficient computational models. However, this powerful processing capability must be subject to constant oversight regarding privacy protection and the appropriateness of computational usage [46]. Human oversight remains essential, both in the decision-making phase regarding the adoption of automated models and in interpreting and contextualizing the data provided. Consequently, AI can become a remarkable tool for physicians and healthcare professionals, enhancing their ability to achieve optimal clinical management.

## 4. Future Perspectives

AI has the extraordinary capability to efficiently, simply, and rapidly analyze vast quantities of data. Thanks to this distinctive characteristic, it is not difficult to envision the pivotal role AI will play in the future medicine.

### 4.1. Precision Medicine

Modern medicine is progressing toward personalized medicine, where diseases are no longer the sole focus of medical intervention. Instead, interventions increasingly consider the unique manifestation of diseases in individual patients, considering their phenotypic characteristics, personal medical history, and family history. Over recent decades, medicine has progressively become precision-centered, placing the patient at the core of clinical practice. AI will undoubtedly play a significant role in enhancing personalized care. Through its powerful data analysis capabilities, healthcare providers will be better-equipped than ever before to quickly and clearly identify patient specific characteristics. This capability will enable clinicians to design highly personalized therapeutic pathways.

### 4.2. Virtual Reality and the Multiverse

In medicine, particularly in cardiology, imaging remains an essential tool for clinicians throughout all stages of patient management, from diagnosis to therapy. Over the years, technological advances have led to the development of increasingly sophisticated and precise imaging techniques. In recent years, imaging itself has undergone significant transformation through the integration of virtual reality (VR).

This emerging technology has the potential to support healthcare professionals not only in routine clinical practice [47] but also in the education of future generations of physicians, who may benefit from increasingly advanced and realistic simulation platforms [48].

Despite the initial enthusiasm, further refinement and investigation are necessary before virtual reality can be reliably implemented in daily clinical practice [49].

In the future, the combination of VR with innovative technologies and the implementation of AI could contribute to the creation of virtual worlds where geographical distances are effectively nullified, enabling routine interactions between professionals even if they are thousands of kilometers apart. In recent years, telemedicine has attracted increasing attention from healthcare professionals, with national healthcare systems recognizing telemedicine to democratize medical access while significantly reducing costs [50]. Supported by advances in connectivity, data sharing, and the development of dedicated virtual environments, AI and telemedicine suggest a future where geographical distance becomes irrelevant, data sharing instantaneous, and healthcare access democratic and universal. Such a future, where telemedicine, virtual reality, and AI form part of everyday clinical practice, could address not only issues related to healthcare access but also facilitate the training of new generations of healthcare professionals. These professionals would benefit from enhanced educational opportunities, allowing them to share knowledge and operative techniques, ultimately transforming medicine from a predominantly national discipline into a truly global cultural practice.

## 5. Conclusions

AI is undoubtedly an important technological advancement, particularly in electrophysiology, where it currently achieves good results in diagnosing arrhythmias and assisting medical personnel during ablation procedures and postoperative planning. Despite these promising outcomes, healthcare professionals remain central to the clinical application of AI in everyday practice. The significant complexity of AI models must be managed through clear regulations to ensure patient privacy. In the future, AI will certainly play a leading role, and when combined with telemedicine and virtual reality, it may push boundaries even further.

## 6. Key Messages

- AI is rapidly emerging as a transformative tool in cardiac electrophysiology, improving diagnostic accuracy in arrhythmia detection, streamlining ECG interpretation, and optimizing catheter ablation procedures through real-time data analysis.
- Deep learning algorithms, particularly CNNs, have demonstrated expert-level or superior performance in identifying complex arrhythmias and stratifying patient risk, even in asymptomatic individuals.
- While the clinical potential of AI is substantial, successful implementation depends on addressing key challenges, including data quality, algorithm transparency, ethical considerations, and ensuring appropriate human oversight in decision-making processes.

## Figures and Tables

**Table 2:** Resume of principal studies, objectives, results, and type of AI architecture in each of the included research. AF: atrial fibrillation; AI: artificial intelligence; ANN: artificial neural network; AUC: area under the curve; CNN: Convolutional Neural Networks; DL: deep learning; ILR: implantable loop recorder; LQTS: long QT syndrome; ML: machine learning; PPV: positive predictive value; PVC: premature ventricular contraction; SCD: sudden cardiac death; SR: sinus rhythm; VT: ventricular tachycardia.

Article	Type of AI	Objectives	Results
IN VITRO/CELLULAR			
Jeong et al. [12]	ANN	To predict changes in cardiac ion channel conductance by analyzing the morphology of simulated action potentials	A mean F1-score of 0.985 and an accuracy of 98.3%, demonstrating strong predictive performance.
Sanchez et al. [13]	ML	To distinguish and characterize non-fibrotic from fibrotic atrial tissue	Strong performance in distinguishing and characterizing atrial tissue
SOPRAVENTRICULAR ARRYTHMIAS			
Hannun et al. [14]	DL	Automated detection and classification of cardiac arrhythmias using single-lead ambulatory electrocardiogram data	AUC of 0.97 and F1 score of 0.837
Attia et al. [15]	CNN	To identify the electrocardiographic signature of AF on standard 10 s, 12-lead ECGs	AUC of 0.87, a sensitivity of 79.0%, a specificity of 79.5%, and an accuracy of 79.4%
Yuan et al. [16]	CNN	To predict the presence of AF within 31 days from outpatient 12-lead ECGs in sinus rhythm	AUC values range from 0.88 to 0.89 and an accuracy of approximately 81–82%
Beak et al. [17]	DL	To detect subtle differences in paroxysmal AF during SR	AUC 0.79 and 0.75 and F1 score of 75% and 74%
Khurshid et al. [18]	CNN	To calculate 5-year AF risk using 12-lead ECGs and compare it to CHARGE-AF score	Similar predictive usefulness of a clinical risk score
Hygrell et al. [19]	CNN	To predict paroxysmal AF from single-lead sinus rhythm electrocardiograms	Mean AUC of 0.71
Mittal et al. [20]	DNN	To evaluate an AI-based solution designed to reduce false-positive atrial fibrillation detections in patients monitored by implantable loop recorders	The PPV of ILR-detected AF episodes increased to 74.5% following use of the AI filter
Sarkar et al. [21]	DL	To reduce inappropriate AF detection by implantable cardiac monitors	Superior specificity compared to existing algorithms
Noseworthy et al. [22]	DL	AI-guided ECG screening during sinus rhythm to detect atrial fibrillation in a real-world population	AI-guided screening was associated with increased detection of atrial fibrillation (high-risk group: 3.6% with usual care vs. 10.6% with AI-guided screening, *p* < 0.0001; low-risk group: 0.9% vs. 2.4%, *p* = 0.12)
Dupulthys et al. [23]	DL	To evaluate a single-lead AI-based ECG model, integrated with clinical risk factors, for detecting atrial fibrillation during sinus rhythm	AUC of 0.74, which increased to 0.76 by adding six risk factors
VENTRICULATARRYTHMIAS			
Yu et al. [24]	DL	Automatic detection of PVCs	Accuracy of 99.7%, sensitivity of 97.45%, and specificity of 99.87%
Missel et al. [25]	Hybrid ML	To localize the origin of ventricular tachycardia using 12-lead ECGs	The model successfully predicted the VT origin with high spatial resolution and outperformed conventional algorithms
Bos et al. [26]	CNN	To identify genetically confirmed patients with LQTS	Maximum AUC of 0.944
Jiang et al. [27]	CNN	Screening and differentiating congenital LQTS	AUC of 0.93 for LQTS detection and an AUC of 0.91 for genotype differentiation
Giudicessi et al. [28]	DNN	To determine QTc in single-lead ECGs	Sensitivity of 80% and specificity of 94.4%
Popescu et al. [29]	DL	To predict the risk of SCD in patients with ischemic heart disease	Concordance index of 0.83 and 0.74 and 10-year integrated Brier score of 0.12 and 0.14
TRANSCATHETER ABLATION			
Seitz et al. [30]	ML	To evaluate VX1 that automatically generate real-time dispersion maps to guide transcatheter ablation	Acute AF termination in 88%. Follow-up showed freedom from AF in 86% after a single procedure, 89% after an average of 1.3 procedures, freedom from any atrial arrhythmia from 54% after one procedure to 73% after repeat procedures (*p* < 0.001)
Di Biase et al. [31]	DL	Anatomical reconstruction of the left atrium from intracardiac echocardiography images	Average anatomical reconstruction time was approximately 65 s
Razeghi et al. [32]	ML	To predict outcomes of AF ablation	High predictive accuracy for AF recurrence post-ablation
Brahier et al. [33]	ML	To identify predisposing factors for AF recurrence	5 covariates were identified as independent predictors of late recurrence
Shade et al. [34]	ML	To predict AF recurrence	Predicted probability of AF recurrence with an average validation sensitivity and specificity of 82% and 89%, respectively, and a validation area under the curve of 0.82

## Data Availability

No new data were created or analyzed in this study. Data sharing is not applicable to this article.
